# Prognosis of Postoperative Cholangitis Following Pancreaticoduodenectomy: A Single-Centered Retrospective Cohort Study

**DOI:** 10.7759/cureus.60392

**Published:** 2024-05-15

**Authors:** Shuhei Yamamoto, Yuki Kataoka, Hanako Kurai, Teiichi Sugiura, Yosuke Yamamoto

**Affiliations:** 1 Division of Infectious Diseases, Shizuoka Cancer Center, Nagaizumi, JPN; 2 Department of Psychosomatic and General Internal Medicine, Kansai Medical University, Hirakata, JPN; 3 Section of Clinical Epidemiology, Department of Community Medicine, Kyoto University Graduate School of Medicine, Kyoto, JPN; 4 Department of Healthcare Epidemiology, Kyoto University Graduate School of Medicine/School of Public Health, Kyoto, JPN; 5 Department of Systematic Reviewers, Scientific Research Works Peer Support Group, Osaka, JPN; 6 Department of Internal Medicine, Kyoto Min-iren Asukai Hospital, Kyoto, JPN; 7 Division of Hepato-Biliary-Pancreatic Surgery, Shizuoka Cancer Center, Nagaizumi, JPN; 8 Department of Healthcare Epidemiology, School of Public Health in the Graduate School of Medicine, Kyoto University, Kyoto, JPN

**Keywords:** retrospective cohort study, prognostic factor, choledochojejunostomy, pancreaticoduodenectomy, postoperative cholangitis

## Abstract

Introduction

Postoperative cholangitis (POC) after pancreaticoduodenectomy is a serious complication. However, the prognostic factors are unclear. We aimed to investigate the relationships between biliary lesions and prognosis in patients with cholangitis after pancreaticoduodenectomy.

Methods

We conducted a single-centered retrospective cohort study. The unit of analysis was hospital admissions. We extracted patients who underwent pancreaticoduodenectomy from 2010 to 2018, and have a record of hospitalization of cholangitis from January 2010 to October 2019. We defined the bile duct lesions as the presence of one of the following: biliary stent, intrahepatic bile duct dilatation, intrahepatic bile duct stones, or common bile duct stones on imaging studies. The primary outcome was the treatment failure of POC. We defined the failure as a composite outcome of death within 30 days of initiation of treatment, relapse during treatment, or recurrence of cholangitis. We used logistic regression analysis to examine the association between the presence of bile duct lesions and the occurrence of outcomes.

Results

Of 154 admissions included in the present study, 120 cases (77.9%) were with bile duct lesions. Bile duct lesions were associated with the treatment failure (crude odds ratio [OR] 2.56, 95% confidence intervals [CI] 1.08 to 6.32; adjusted OR 2.81, 95%CI 1.08 to 7.34).

Conclusions

Clinicians should follow the patient of POC with bile duct lesions on imaging carefully because of the high risk of treatment failure, especially for recurrence. Further studies are warranted to confirm our results.

## Introduction

Cholangitis is a serious complication that can occur following surgical procedures involving the biliary tract as represented by pancreatoduodenectomy [1]. Improved perioperative prognosis has focused attention from perioperative to postoperative cholangitis (POC) [2,3]. The exact epidemiology of postoperative cholangitis is not well understood, but it is thought to occur in 10%-17% of patients who have undergone pancreatoduodenectomy. Risk factors for the development of POC include male gender, postoperative hepatolithiasis, and postoperative anastomotic stricture [1].

Although there are several studies on the risk of developing POC [1,4], studies on prognostic factors are scarce. A case series including 19 patients of POC suggested that anastomotic stenosis may be a risk factor for recurrent cholangitis [2]. However, no study has fully investigated the relationships between the bile duct lesion and prognosis in patients with POC.

We conducted a retrospective cohort study aimed at investigating the relationships between biliary lesions and prognosis in patients with POC after pancreaticoduodenectomy.

## Materials and methods

Study design and participants

We performed a hospital-based retrospective cohort study. This study was conducted in Shizuoka Cancer Center Hospital in Japan where approximately 100 pancreaticoduodenectomies are performed annually. We reported this study following the Strengthening the Reporting of Observational Studies in Epidemiology (STROBE) statement (https://osf.io/y4z35/) [5].

Patients

The unit of analysis was hospital admissions. We extracted patients who underwent pancreaticoduodenectomy from 2010 to 2018, and have a record of hospitalization of cholangitis from January 2010 to October 2019. If the same patient was newly hospitalized for another cholangitis, the patient was counted as a different case. In contrast, if a patient was re-admitted due to the relapse of cholangitis, we considered the hospitalization as the same episode.

We included all the admissions with administrative records of cholangitis; ICD-10 codes were retrograde cholangitis K83.0, acute suppurative cholangitis K83.0, acute cholangitis K83.0, recurrent cholangitis K83.0, cholangitis K83.0, acute obstructive pyogenic cholangitis, K83 .0, postoperative cholangitis K83.0 (ICD10-2), or K91.8 (ICD10-1). We excluded cases for preoperative cholangitis, infections other than cholangitis (such as urinary tract infection, pyelonephritis, prostatitis, catheter-related bloodstream infection, pneumonia, meningitis, cellulitis, peritonitis, febrile neutropenia, pancreatitis), or biliary obstruction without cholangitis. We also excluded cholangitis with liver abscess at onset in order to determine the prognosis of cholangitis with and without bile duct involvement, but included cases in which liver abscess developed during treatment for cholangitis. To exclude non-cholangitis admissions, we used administrative data and one board-certified infectious disease physician (SY) confirmed the diagnosis based on electronic medical charts.

We used two criteria for diagnosing POC. The first criterion was the clinical diagnosis. We diagnosed patients as POC when treated clinically as cholangitis, with elevated hepatobiliary enzymes, but excluded those without elevated hepatobiliary enzymes. The second criterion was the diagnostic criteria of Tokyo Guidelines 2018 (TG18) [6]. Among administrative-records-based cholangitis admissions, we adopted TG18 and diagnosed as definite cases, suspected cases, and possible cases. One board-certified infectious disease physician (SY) performed the diagnosis process based on electronic medical charts.

Exposures

We compared the included cases according to the presence or absence of bile duct lesions. We defined the bile duct lesions as the presence of one of the following: biliary stent, intrahepatic bile duct dilatation, intrahepatic bile duct stones, or common bile duct stones on imaging studies such as abdominal CT, abdominal echocardiography, PET-CT, or MRI during the 7 days before and after the start of cholangitis treatment. We adopted imaging report findings by the radiologist. If no report was available or the report findings could not be used to make a decision, the decision was based on the actual imaging findings and the medical chart by the treating physician.

Outcome measurements

The primary outcome was the treatment failure of POC. We defined the failure as a composite outcome of death within 30 days of initiation of treatment, relapse during treatment, or recurrence of cholangitis. We set each of the composite outcome items as a secondary outcome: death within 30 days of starting treatment, relapse during treatment, and recurrence more than one day after treatment ended until 90 days after the start of treatment for the previous POC.

Data collection

We extracted case information from the electronic medical charts and administrative data. We summarized the detailed definitions of the variables (https://osf.io/y4z35/). Bile cultures were not included in this study because they were collected only in patients with drainage.

Statistical analysis

We used summary statistics to describe patient characteristics. For categorical variables, we used Fisher's exact test and logistic regression analysis to examine the association between the presence or absence of bile duct lesions and the occurrence of primary and secondary outcomes, with adjustment for possible confounders mentioned below. In the protocol, we predetermined confounding variables as age, postoperative malignant status, cytotoxic anticancer agents within 2 months, Charlson comorbidity index, perioperative (whether within 30 days from surgery), severity of POC by TG18, multidrug-resistant bacteria, resistance to the first antibiotics, initial treatment with oral antibiotics, and drainage. We treated missing values of laboratory data as the normal category to classify the severity, because most patients without measurement of laboratory data were presumed to have mild symptoms. For sensitivity analysis, we limited only definite and suspected patients according to the TG18 diagnostic criteria. We also conducted complete-case analysis for severity. We used Posit Cloud for the analysis which is a cloud-based integration development environment using R [7]. A p-value of < 0.05 was assumed to be statistically significant.

Ethical considerations

The institutional review board at the Shizuoka Cancer Center approved a waiver of informed consent by information disclosure (J2019-78-2019-1-3).

## Results

Study population

**Figure 1:**
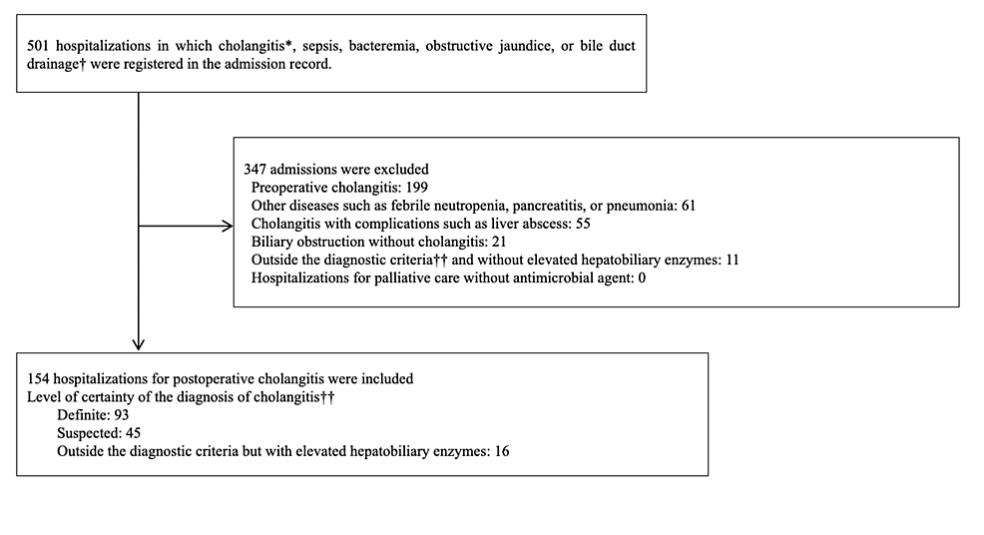
Patients flow chart * Cholangitis includes the following diseases: cholangitis, retrograde cholangitis, acute suppurative cholangitis, acute cholangitis, stenosing cholangitis, recurrent cholangitis, obstructive suppurative cholangitis, and postoperative cholangitis as recorded disease names. † Bile duct drainage includes the following procedures: external biliary drainage, percutaneous transhepatic biliary drainage, percutaneous transhepatic biliary stenting, endoscopic nasobiliary drainage, endoscopic biliary dilatation, endoscopic biliary stenting, endoscopic ultrasound-guided biliary drainage. †† The Diagnostic criteria for cholangitis are based on the Tokyo Guidelines 2018. In cases clinically diagnosed as cholangitis but outside the diagnostic criteria, those with elevated biliary enzymes were included, and those without elevated biliary enzymes were excluded.

From 2010 to 2018, 882 patients underwent pancreaticoduodenectomy. Of these, 346 patients had a total of 501 admissions which had records of cholangitis, sepsis, bacteremia, obstructive jaundice, or bile duct drainage (Figure 1). After excluding 347 admissions, we included 154 admissions of 120 patients due to POC. Among them, 138 admissions met TG18 diagnostic criteria (Definite: 93, Suspected: 45).

We summarize the case characteristics in Table 1 and Table 2. Out of 154 cases, 120 cases (77.9%) were with bile duct lesions. The reason for the pancreaticoduodenectomy was malignancies in 145 cases (94.1%). A total of 134 cases (94.1%) were not in perioperative periods. The mean antimicrobial treatment days were 10.68 days (standard deviation [SD] 11.85) with bile duct lesions and 11.53 days (SD 13.11) without bile duct lesions.

**Table 1:** Case characteristics due to postoperative cholangitis N: number, SD: standard deviation, TG18: Tokyo Guideline 2018

	Presence of bile duct lesions (N=120)	Absence of bile duct lesions (N=34)	Total (N=154)
Age, years (mean [SD])	69.56 [10.04]	69.62 (7.80)	69.57 [9.56]
Gender, males (%)	90 (75.0)	27 (79.4)	117 (76.0)
Details of Bile duct lesion (with duplications)			
Biliary dilatation (%)	113 (94.2)		
Stone (%)	14 (11.7)		
Stent (%)	28 (23.3)		
Pancreaticoduodenectomy due to malignancy (%)	113 (94.1)	32 (94.1)	145 (94.1)
Site of lesion			
Pancreas (%)	68 (56.7)	16 (47.1)	84 (54.5)
Biliary tract (%)	37 (30.8)	8 (23.5)	45 (29.2)
Duodenum (%)	13 (10.8)	9 (26.5)	22 (14.3)
Others (%)	2 (1.7)	1 (2.9)	3 (1.9)
Malignancy status			
Cure or non-cancer (%)	79 (65.8)	27 (79.4)	106 (68.8)
Recurrence or residual (%)	41 (34.2)	7 (20.6)	48 (31.2)
Cytotoxic chemotherapy within 2 months (%)	21 (17.5)	5 (14.7)	26 (16.9)
Charlson comorbidity index (mean [SD])	1.21 [1.14]	1.24 [0.89]	1.21 [1.09]
Non-perioperative admissions (%)	103 (85.8)	31 (91.2)	134 (87.0)
TG18 severity criteria			
Grade 1 (mild)	38 (31.7)	9 (26.5)	47 (30.5)
Grade 2 (moderate)	65 (54.2)	19 (55.9)	84 (54.5)
Grade 3 (severe)	17 (14.2)	6 (17.6)	23 (14.9)
Start with oral antibiotics	20 (16.7)	0 (0.0)	20 (13.0)
Resistance to the first antibiotics	19 (15.8)	6 (17.6)	25 (16.2)
Duration of antimicrobial treatment, days (mean [SD])	10.68 [11.85]	11.53 [13.11]	10.87 [12.10]
Biliary drainage (%）	43 (35.8)		

**Table 2:** detailed admission characteristics N: number, SD: standard deviation, WBC: white blood cell, PLT: platelet, PT-INR: Prothrombin Time-International Normalized Ratio, AST: aspartate aminotransferase, ALT: alanine aminotransferase, ALP: alkaline phosphatase, γ-GTP: γ-Glutamyl transpeptidase, T-Bil: total bilirubin, Cr: creatinine, Alb: albumin, CRP: C-reactive protein, TG18: Tokyo Guideline 2018, ERCP: Endoscopic retrograde cholangiopancreatography, PTCD: Percutaneous transhepatic cholangio drainage, EUS-BD: Endoscopic ultrasound-guided biliary drainage

	Presence of bile duct lesions (N=120)	Absence of bile duct lesions (N=34)	Total (N=154)
Barthel index (mean [SD])	98.08 (11.51)	100.00 (0.00)	98.51 (10.18)
Body temperature (mean [SD])	38.31 [1.01]	38.54 [0.92]	38.36 [0.99]
missing (%)	1 (0.8)	0 (0.0)	1 (0.64)
Cardiovascular dysfunction (%)	1 (0.8)	0 (0.0)	1 (0.64)
Neurological dysfunction (%)	0 (0.0)	0 (0.0)	0 (0.0)
Respiratory dysfunction (%)	4 (3.3)	1 (2.9)	5 (3.2)
WBC (mean [SD])	9258.67 [3927.55]	10025.29 [4846.42]	9427.92 [4143.13]
PLT (mean [SD])	22.12 [10.70]	16.55 [6.04]	20.89 [10.11]
PT-INR (mean [SD])	1.32 [0.65]	1.10 [0.08]	1.26 [0.56]
missing (%)	90 (75.0%)	23 (67.6%)	113 (73.4)
AST (mean [SD])	100.67 [140.38]	114.06 [114.92]	103.63 [134.93]
ALT (mean [SD])	73.14 [72.79]	108.47 [140.84]	80.94 [92.82]
ALP (mean [SD])	1024.50 [871.27]	594.97 [261.34]	928.42 [797.10]
missing (%)	2 (1.7)	0 (0)	2 (13.3)
γ-GTP (mean [SD])	341.57 [378.95]	161.77 [144.53]	299.07 [346.46]
missing (%)	36 (30)	8 (23.5)	44 (28.6)
T-Bil (mean [SD])	1.61 [1.67]	1.16 [0.52]	1.51 [1.50]
Cr (mean [SD])	0.76 [0.19]	0.89 [0.24]	0.78 [0.21]
Alb (mean [SD])	3.20 [0.61]	3.44 [0.56]	3.26 [0.61]
missing (%)	2 (1.7)	0 (0)	2 (13.3)
CRP (mean [SD])	6.06 [4.84]	5.28 [4.15]	5.89 [4.69]
missing (%)	1 (0.83)	0 (0)	1 (0.64)
TG18 diagnostic criteria			
Systemic inflammation (%)	115 (95.8)	33 (97.1)	148 (96.1)
Cholestasis (%)	96 (80.0)	23 (67.6)	119 (77.3)
Bile duct lesion (%)	120 (100.0)	0 (0.0)	120 (77.9)
Techniques of drainage			
ERCP	21		
PTCD	14		
Cleaning of existing bile duct tubes	7		
EUS-BD	1		
Diagnosis			
Suspected cases without the criteria	5 (4.2)	11 (32.4)	16 (10.4)
Suspected cases within the criteria	22 (18.3)	23 (67.6)	45 (29.2)
Definite cases	93 (77.5)	0 (0.0)	93 (60.4)
Start treatment with antipseudomonal antibiotics	82 (68.3)	22 (64.7)	104 (67.5)

Blood culture

We present the results of the blood culture tests in Table 3. Blood cultures were obtained from 91 of 120 (75.8%) cases with bile duct lesions, and 27 of 34 (79.4%) cases without bile duct lesions. A total of 38 (41.7%) hospitalizations with bile duct lesions and 11 (40.7%) hospitalizations without bile duct lesions had positive blood cultures. In patients with bile duct lesions, *Escherichia coli *(16, 18%), *Klebsiella* spp. (13, 14%), and *Aeromonas *spp. (4, 4%) were the most common bacteria identified, while in those without lesions, *Escherichia coli *(5, 19%),* Klebsiella *spp. (2, 7%), and *Aeromonas *spp. (3, 11%) prevailed. Multidrug-resistant bacteria were identified in seven (8%) with bile duct lesions and three (11%) without bile duct lesions.

**Table 3:** Characteristics of blood cultures N: number, SD: standard deviation, ESBL: extended-spectrum beta-lactamases

	Presence of bile duct lesions N = 91	Absence of bile duct lesions N = 27	All admissions obtained blood cultures N = 118
Escherichia coli	16 (18%)	5 (19%)	21 (18%)
*Klebsiella *spp.	13 (14%)	2 (7%)	15 (13%)
*Aeromonas* spp.	4 (4%)	3 (11%)	7 (6%)
*Enterococcus* spp.	5 (5%)	1 (4%)	6 (5%)
*Streptococcus* spp.	0 (0%)	2 (7%)	2 (2%)
*Acinetobacter* spp.	1 (1%)	0 (0%)	1 (1%)
*Citrobacter* spp.	1 (1%)	0 (0%)	1 (1%)
Clostridium perfringens	1 (1%)	0 (0%)	1 (1%)
Edwardsiella tarda	1 (1%)	0 (0%)	1 (1%)
*Serratia* spp.	1 (1%)	0 (0%)	1 (1%)
Stenotrophomonas maltophilia	1 (1%)	0 (0%)	1 (1%)
Polymicrobials	6 (7%)	2 (7%)	8 (7%)
Multidrug-resistant bacteria	7 (8%)	3 (11%)	10 (8%)
ESBL-producing bacteria	4 (4%)	1 (4%)	5 (4%)

Outcomes

In univariate analysis, 66 (55%) cases with bile duct lesions and 11 (32.3%) cases without bile duct lesions had treatment failure (odds ratio [OR] 2.56, 95% confidence intervals [CI] 1.08 to 6.32). (Table 4). A total of 47 (39.2%) cases with bile duct lesions and nine (26.5%) admissions had recurrence (OR 1.79, 0.73 to 4.73). 

**Table 4:** Outcomes of postoperative cholangitis admissions N: number, OR (95%CI): Crude odds ratio by the presence of bile duct lesions and 95% confidence intervals

	Presence of bile duct lesions (N=120)	Absence of bile duct lesions (N=34)	All cases (N=154)	OR (95%CI)
Treatment failure	66 (55.0)	11 (32.4)	77 (50.0)	2.56 (1.08 to 6.32)
Recurrence	47 (39.2)	9 (26.5)	56 (36.3)	1.79 (0.73 to 4.73)
Relapse	11 (9.2)	2 (5.9)	13 (8.4)	1.61 (0.33 to 15.69)
Mortality	8 (6.7)	0 (0.0)	8 (5.2)	Unestimable

In multivariable logistic regression analysis, the risk of treatment failure was higher in those with bile duct lesions with an adjusted odds ratio of 2.81 (95%CI 1.08 to 7.34) (Table 5). In sensitivity analysis, limited only definite and suspected patients according to the TG18 diagnostic criteria showed an adjusted OR of 2.73 (95%CI 0.89 to 8.37). Complete case analysis excluding the cases imputed severity showed an adjusted OR of 2.9 (95%CI 0.13 to 63.7).

**Table 5:** The relationship between bile duct lesion and prognosis. OR: odds ratio, CI: confidence intervals Sensitivity analysis 1: limited only definite and suspected cases according to the TG18 diagnostic criteria Sensitivity analysis 2: excluding the cases imputed severity

	crude OR	95% CI	adjusted OR	95% CI
Main analysis	2.56	1.14 to 5.71	2.81	1.08 to 7.34
Sensitivity analysis 1	2.27	0.89 to 5.78	2.73	0.89 to 8.37
Sensitivity analysis 2	2.8	0.56 to 13.95	2.9	0.13 to 63.7

## Discussion

We conducted a retrospective cohort study including 154 admissions of 120 patients after pancreaticoduodenectomy POC. Among them, 77.9% had bile duct lesions and 8% had a positive blood culture of multidrug-resistant bacteria. Regardless of with or without bile duct lesions, they received approximately 11 days of antibiotic treatment. Among patients without bile duct lesions, 26.5% patients suffered recurrence. We found that patients with bile duct lesions had a worse prognosis. The relationship was robust in the sensitivity analyses. 

This is the first study to investigate the bile duct lesions as a prognostic factor in POC. A previous review indicated that postoperative hepatolithiasis and postoperative anastomotic stricture are related to the higher incidence of POC after biliary-enteric anastomosis [1]. Our results indicate that bile duct lesions are not only a risk factor for POC but also a prognostic factor. Clinicians should monitor and manage patients with bile duct lesions closely, keeping in mind the possibility of multidrug-resistant organisms. 

Our results also indicate that the short-term prognosis of patients without bile duct lesions was better than patients with bile duct lesions, however, the recurrence is worse than source-controlled cholangitis [8]. Tokyo Guidelines 2018 recommends antimicrobial therapy for 4 to 7 days when the source of infection is controlled. Such patients seldom suffer recurrence [9,10]. To determine the appropriate duration of treatment for patients without bile duct lesions to prevent relapse, further cohort studies are needed to explore the relationship between the duration of antimicrobial treatment and relapse in postoperative patients without bile duct lesions.

There are several limitations in our study. First, we excluded patients only treated in the clinic. This exclusion may have selectively excluded patients without bile duct lesions, however, this exclusion may weaken the relationship, because such patients will have a better prognosis. Second, this study was conducted in a high-volume center in Japan, however, due to the sample size limitation, we could not sufficiently estimate the adjusted odds ratios of secondary outcomes. Further large multi-centered studies to confirm our findings are warranted. Third, we adopted a clinical diagnosis of POC for the main analysis. TG18 diagnostic criteria is a reference standard, but not necessarily accurate [11,12]. In addition, the point estimates were not so different from the sensitivity analysis limiting the patients to those who met the TG18 diagnosis. Fourth, the lack of quality control in some subjective measurements would cause misclassification. Further measurement improvements are needed in future studies.

## Conclusions

We investigated the relationships between biliary lesions and prognosis in patients with cholangitis after pancreaticoduodenectomy. Our results indicate that clinicians should follow the patient of POC with bile duct lesions on imaging carefully, because of the high risk of treatment failure, especially for recurrence. In addition, further cohort studies are warranted to determine the optimal duration of antimicrobial treatment in postoperative patients without bile duct lesions to prevent relapse.
